# Efficacy and cost-effectiveness analysis of 10-day versus 14-day eradication of *Helicobacter pylori* infection with vonoprazan amoxicillin: a prospective, multicenter, randomized controlled trial

**DOI:** 10.3389/fphar.2025.1543352

**Published:** 2025-03-24

**Authors:** Yunfan Dong, Zhaotao Duan, Min Liu, Yanbing Ding, Guangxia Chen, Ruifang Wang, Xiaodan Xu, Lixia Ding, Qiang Zhan, Chengyu Pan, Hui Li, Faming Yang, Xiaorong Dai, Xiangsu Li, Xudong Wu, Peng Peng, Jianrong Wang, Kewei Hu, Duanmin Hu, Qiong Jie, Zhenyu Zhang

**Affiliations:** ^1^ Department of Gastroenterology, Nanjing First Hospital, Nanjing Medical University, Nanjing, China; ^2^ Department of Gastroenterology, Affiliated Hospital of Yangzhou University, Yangzhou University, Yangzhou, China; ^3^ The First People’s Hospital of Xuzhou, Xuzhou Municipal Hospital Affiliated to Xuzhou Medical University, Xuzhou, China; ^4^ Department of Gastroenterology, Changshu No.1 People’s Hospital, Suzhou, China; ^5^ Departments of Gastroenterology, Affiliated Wuxi People’s Hospital of Nanjing Medical University, Wuxi, Jiangsu, China; ^6^ Affiliated Hospital of Integrated Traditional Chinese and Western Medicine, Nanjing University of Chinese Medicine, Nanjing, China; ^7^ Department of Gastroenterology, Taixing People’s Hospital, Taixing, China; ^8^ Yancheng No.1 People’s Hospital, Affiliated Hospital of Medical School, Nanjing University, Yancheng, China; ^9^ Department of Gastroenterology, Taizhou Fourth People’s Hospital, Taizhou, China; ^10^ Department of Gastroenterology, The Second Affiliated Hospital of Soochow University, Suzhou, China; ^11^ Department of Pharmacy, Nanjing First Hospital, Nanjing Medical University, Nanjing, China

**Keywords:** *Helicobacter pylori*, vonoprazan, amoxicillin, dual therapy, eradication, cost-effectiveness

## Abstract

**Objectives:**

To evaluate the efficacy and cost-effectiveness of 10-day vonoprazan-amoxicillin (VA) dual therapy compared to 14-day VA therapy.

**Methods:**

A non-inferiority trial was carried out at 10 clinical centers to recruit patients with H. pylori infection. Subjects were assigned at random to either the group for 10-day or 14-day, and where given vonoprazan 20 mg bid and amoxicillin 1 g tid. Comparisons were made in terms of eradication rates, adverse events, cost-effectiveness, and compliance.

**Results:**

914 participants were enrolled and randomly assigned to either the 10-day or 14-day VA groups. Using the intention-to-treat principle and multiple imputation for missing outcomes, the analysis showed an eradication rate of 88.79% in the 10-day group and 92.37% in the 14-day group (P = 0.064). The eradication rates were 89.14% and 93.35% by per-protocol analysis (P = 0.037). There were no significant differences in adverse events or compliance between the groups (P > 0.05). Logistic regression analysis indicated that smoking and prior failure of eradication were risk factors influencing the eradication rate (P < 0.05). For the economic evaluation, the cost-effectiveness ratio (CER) of the 10-day group was 426.30 yuan, the CER of the 14-day group was 485.27 yuan, and the incremental cost-effectiveness ratio was 1680.23 yuan. In probability sensitivity analysis, the cost-effectiveness acceptability curve showed that when the willingness-to-pay(WTP) threshold was below 1742 yuan, the 10-day group was more cost-effective. When the WTP threshold was above 1742 yuan, the 14-day group was more cost-effective.

**Conclusion:**

In this study, the 10-day VA was not found to be inferior to the 14-day VA. Compared with the 14-day group, the 10-day group is more cost-effective, but as the WTP threshold increases to 1742 yuan, the probability of the 14-day group being more cost-effective was greater than that of the 10-day group. Smoking and previous eradication attempts were associated with the eradication failure of VA therapy.

**Clinical Trial Registration:**

https://clinicaltrials.gov/, identifier NCT05469685.

## Introduction

Approximately half of the world’s population carries *H. pylori* (*H. pylori*), a harmful bacteria that lives in the stomach. It is linked to a range of upper gastrointestinal conditions, such gastritis, ulcers in the digestive tract, and stomach cancer. Due to its high prevalence and harmfulness, it brings huge cost burden to the social medical resources.

The Maastricht Consensus Report proposed the most classic eradication treatment for *H. pylori* globally, consisting of a triple therapy combining PPI, amoxicillin, and either clarithromycin or metronidazole (Current European concepts in the management of Helicobacter pylori infection. The Maastricht Consensus Report, 1997). Nevertheless, the global rise in antibiotic resistance among *H. pylori* has led to a decrease in the success rate of triple therapy below the necessary level for medical standards. As a result, the bismuth quadruple therapy (BQT) was suggested as a viable treatment option by the Maastricht IV Consensus in 2012 (Malfertheiner et al., 2012). And it has gradually become the first-line treatment in China. Despite its efficacy, BQT is associated with certain limitations, including complicated medication regimen, adverse reactions, and poor patient compliance.

Over the past 10 years, research has shown that combining high doses of proton pump inhibitors (PPIs) with high doses of amoxicillin(HDDT) can effectively eradicate *H. pylori* with fewer side effects compared to BQT in various studies (Macedo et al., 2023; Zhou et al., 2023). Studies have shown that the 14-day HDDT regimen is not inferior to the BQT, with good treatment compliance, and is also safe and effective in elderly patients or rescue treatment (Yang et al., 2023; Bi et al., 2022). In spite of this, a propensity score matching analysis showed that the success rate of the 10-day HDDT was just 80%, which suggests a lack of efficacy (Zou et al., 2021).

With the advent of more effective acid-blocking agents than PPIs, vonoprazan-based dual therapy was regarded as a promising new first-line option. In Japan, 7-day vonoprazan-amoxicillin(VA) dual therapy has achieved good eradication rate (Suzuki et al., 2020; Gotoda et al., 2020).

In China, a study conducted by Lin et al. showed that the success rate of eliminating *H. pylori* with the 7-day VA therapy was below 70%, contradicting the favorable outcomes seen in Japanese research (Lin et al., 2022). This discrepancy could be due to variations in the prevalence and resistance of *H. pylori* strains. While the 14-day VA has good eradication rates in China and has potential as a first-line and rescue treatment (Hu et al., 2022a; Gao et al., 2022). There is currently debate surrounding the effectiveness of the 10-day VA treatment. The 10-day VA regimen achieved an eradication rate of 81.1%, as Hu et al. reported (Hu et al., 2022b). However, Qian et al. demonstrated a 93.4% success rate in eliminating the infection with the 10-day high-dose VA therapy (Qian et al., 2023). Due to the limited research on the 10-day VA therapy in China, additional investigation is needed.

A multicenter, open-label, randomized controlled trial was carried out to evaluate the effectiveness and safety of 10-day compared to 14-day VA treatment for *H. pylori* infection, and a cost-effectiveness evaluation was also performed, aiming to establish the most suitable duration.

## Methods

### Study design

The study was conducted in ten medical institutions in China from August 2022 to August 2023 and was registered under the identifier NCT05469685 on ClinicalTrials.gov. The research followed the guidelines of the Helsinki Declaration and was approved by the institutional review board at all participating locations. All participants provided written consent prior to the study.

### Subjects

Detailed inclusion criteria include (1) Being between 18 and 65 years old; (2) Having a confirmed *H. pylori* infection through the ^13^C-urea breath test; (3) Not having received eradication therapy in the last 6 months; (4) Being willing to sign the informed consent form.

The study excluded patients who did not meet the specified criteria. (1) Allergic to the study drugs; (2) Presence of active peptic ulcer; (3) Use of antibiotics or bismuth agents within the past 4 weeks or use of histamine H2 receptor antagonists, PPIs, or vonoprazan within the past 2 weeks prior to study; (4) Chronic administration of adrenal corticosteroids, nonsteroidal anti-inflammatory drugs, or anticoagulants; (5) History of esophageal or gastric surgery; (6) Pregnancy or lactation; (7) Presence of severe comorbid diseases, including liver, cardiovascular, lung, and kidney disorders; (8) Heavy drinkers; (9) The existence of mucosa-associated lymphoid tissue lymphoma (MALT) and cancerous tumors.

All enrolled women of childbearing age are provided with education, and appropriate contraceptive measures are recommended during the study period.

### Sample size

Drawing from previous studies on VA therapy, we assumed a 90% eradication rate in the 10-day VA group and a 93.5% eradication rate in the 14-day VA group. With a two-sided alpha level of 0.05, which is the same as a one-sided alpha level of 0.025, a statistical power of 0.9 (1-beta), and a 1:1 ratio of sample sizes in both groups, as well as a non-inferiority margin of −0.1, the sample size was determined to be 375 for each the experimental and control groups. Taking into account a potential follow-up and dropout rate of 15%, the final minimum sample size required rises to 442 for both groups, for a total of 884 cases.

### Randomization and intervention

Participants meeting the eligibility criteria were randomly assigned to either the 10-day or the 14-day VA group in a 1:1 ratio based on a randomized allocation sequence. This randomization process was carried out by professional biostatisticians using SAS 9.4 statistical analysis system programming. A stratified block randomization method was employed, stratified by research centers, and the block length was set as 4.

After signing the informed consent form, eligible subjects were required to fill out electronic questionnaires on the professional online questionnaire survey platform “Questionnaire Star” (Changsha Ransheng Information Technology Co., Ltd.). Information on demographics and clinical data was collected, including gender, age, BMI, smoking, alcohol drinking, gastrointestinal symptoms, and history of antibiotic use within the past 2 years. A random allocation sequence generated by professional statisticians was input to the questionnaire platform backstage before the start of the study and matched with the questionnaire serial numbers generated based on the time order of questionnaire submission. Patients who were enrolled were randomly assigned to one of the two treatment groups based on the order in which they submitted the questionnaire.

Depending on their assigned group, the participants were given either 10-day or 14-day VA therapy, with vonoprazan 20 mg twice daily and amoxicillin 1000 mg three times daily.

### Outcomes

The primary endpoint was the *H. pylori* eradication rate based on a negative ^13^C-urea breath test 4–8 weeks after the end of eradication therapy. The negative ^13^C-urea breath test was determined by a DOB value below 4.

The secondary endpoints included adverse events and drug compliance. Adverse events, graded using a four-point scale designed to evaluate the severity, were classified as none, mild (discomfort that did not interfere with normal activities), moderate (sufficient discomfort to cause interference with normal activities), or severe (severe discomfort that required discontinuation of therapy) (Bi et al., 2022). Adverse events were collected through WeChat and phone during the first week and at the end of eradication treatment (14 days). Drug compliance was assessed by the number of times the subjects correctly took the medication at the end of eradication therapy (14 days), and a compliance rate of more than 80% was considered good.

### Cost-effectiveness analysis

Cost: This study was based on the perspective of the Chinese health system and analyzes direct medical costs, including drug costs, examination costs (registration fees, breath test C13), and costs of handling adverse events. That is, the total direct medical cost = drug cost+examination cost+adverse events cost. All expenses were calculated based on the 2023 Jiangsu Provincial Medical Fee Standards. Vonoprazan Takeda Pharmaceutical Company Limited (Walker, 20mg × 7 tablets/box) costs 69.3 yuan, amoxicillin capsules (Zhejiang Jinhua Kangenbei Pharmaceutical Co., Ltd., Jinkang, 0.25g × 24 capsules/box) costs 1.90 yuan, registration fee was 12 yuan, and ^13^C-urea breath test costs 150 yuan; The cost of handling adverse events was obtained through telephone follow-up. Due to the short duration of this study, the discounting issue was not considered.

Evaluation methods: Pharmacoeconomic evaluation methods were divided into cost-minimization, cost-effectiveness, cost-utility and cost-benefit analyses (Arenas-Guzman et al., 2005). This study used the cost-effectiveness method based on the outcome indicators of the clinical trial.

Sensitivity analysis: In pharmacoeconomic evaluations, due to the uncertainty of data collection and research assumptions that could affect research results, further sensitivity analysis was needed to explore the robustness of basic analysis. This study used the non-parametric Bootstrap probability sensitivity analysis method to evaluate the robustness of basic analysis. Two sets of samples with the same number were selected from the original sample based on replacement sampling, and 1000 simulations were conducted to obtain point estimates for multiple samples. Based on the analysis results, an incremental cost-effectiveness scatter plot was drawn, and a cost-effectiveness acceptability curve (CEAC) was drawn by combining different willingness-to-pay thresholds. When making decisions, compare the incremental cost-effectiveness ratio with the decision-maker’s willingness-to-pay threshold to evaluate whether the plan was cost-effective. If the incremental cost-effectiveness ratio was lower than the decision maker’s willingness-to-pay threshold, then this scheme was considered cost-effectiveness compared to another scheme; If the incremental cost-effectiveness ratio was higher than the decision maker’s willingness-to-pay threshold, then this scheme was considered uneconomical compared to another scheme (Arenas-Guzman et al., 2005). The willingness-to-pay threshold in this study referred to the highest price decision-makers were willing to pay for a 1% increase in eradication rate. Due to the fact that the outcome indicator of this study was an effect indicator rather than a utility indicator, and there was currently no unified willingness-to-pay threshold, this study assumes that the willingness-to-pay threshold was compared between 0 and 100000 yuan, reflecting the economic probability of the 10 day and 14-day groups under different willingness-to-pay thresholds. CEAC demonstrated the probability of economic viability for the 10-day and 14-day groups under different payment intention thresholds.

### Statistical analysis

The Full Analysis Set (FAS) consists of all participants who were randomly assigned and took at least one dose of the experimental medication. c. Missing data were imputed based on the ITT principle using multiple imputation, using all available predictor variables to estimate missing data values, and creating 5 copies of the data. Multiple imputation was performed using SPSS software based on all relevant variables except for family history of gastric cancer (due to the small number of subjects with family history of gastric cancer). The Per Protocol Set (PPS) included subjects who had good compliance with the trial protocol and underwent ^13^C-urea breath test after treatment. Statistical analyses were performed using SPSS software. The chi-square test or Fisher’s exact test was used to analyze categorical variables. If the continuous data followed a normal distribution, it presented as the mean ± standard deviation (SD) and t-test were used. If the continuous data did not follow a normal distribution, it presented as the median (interquartile range) and Wilcoxon rank-sum test were used. P values <0.05 were considered statistically significant. Non-inferiority testing utilized the discrepancy in eradication rates between the 10-day and 14-day treatment plans, in addition to the 95% confidence interval (CI). The non-inferiority of 10-day group to 14-day group was assessed by one-sided non-inferiority Z test. The 95% CI of difference between the two groups in eradication rates were calculated. Non-inferiority would be concluded if the P value of non-inferiority Z test was less than 0.025 or the lower limit of 95% CI for the difference between the two groups was more than −10%. Logistic regression analysis was used to identify potential factors influencing the primary outcome. Excel 2019 software was used for non-parametric Bootstrap sensitivity analysis.

## Results

### Baseline characteristics

**FIGURE 1:**
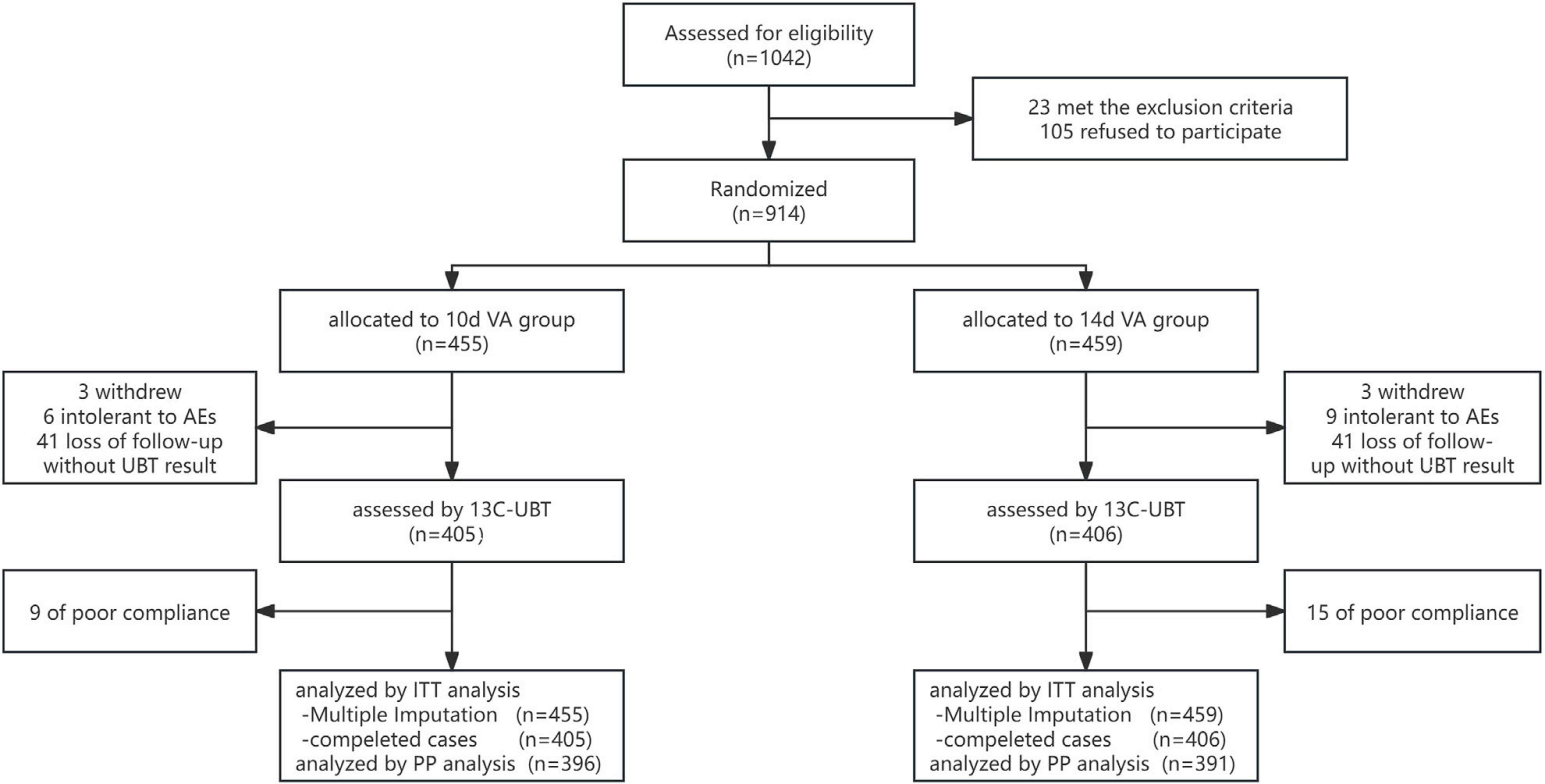
Flow diagram of this study. VA, vonoprazan-amoxicillin dual therapy, ITT, intention-to-treat, PP, per-protocol, UBT, urea breath test, AEs, adverse events.

Figure 1 displays the screening of 1042 patients with *H. pylori* infection, resulting in the recruitment of 914 suitable participants who were randomly divided into two groups receiving different treatment durations: 10-day or 14-day VA group. The demographic and clinical features of the Intent-to-Treat (ITT) population are thoroughly outlined in Table 1. Analysis showed that there were no notable variations in gender, age, Body Mass Index (BMI), smoking habits, gastrointestinal symptoms, history of *H. pylori* eradication, family infection status of *H. pylori*, family history of gastric cancer, and use of antibiotics in the previous 2 years (P > 0.05).

**TABLE 1:** Patient demographics and baseline characteristics in 10d VA group and 14d VA group.

	10d VA group n = 455	14d VA group n = 459	P
Age	43.86±12.44	42.53±12.37	0.104
BMI	23.30 ± 3.09	23.26 ± 3.12	0.862
Sex			
Male	210 (46.15%)	207 (45.10%)	0.749
Female	245 (53.85%)	252 (54.90%)	
Education			
High school or below	178 (39.12%)	174 (37.91%)	0.706
College or above	277 (60.88%)	285 (62.09%)	
Comorbidity			
Hypertension	64 (14.07%)	56 (12.20%)	0.404
Heart disease	5 (1.10%)	7 (1.53%)	0.571
Diabetes	15 (3.30%)	17 (3.70%)	0.738
Fatty liver	47 (10.33%)	59 (12.85%)	0.233
Smoking	79 (17.36%)	77 (16.78%)	0.814
Alcohol drinking	132 (29.01%)	134 (29.19%)	0.951
Digestive symptoms	209 (45.93%)	191 (41.61%)	0.188
Abdominal pain	62 (13.63%)	71 (15.47%)	0.430
Abdominal distension	101 (22.20%)	117 (25.49%)	0.243
Abdominal discomfort	58 (12.75%)	81 (17.65%)	0.039
Acid reflux	100 (21.98%)	95 (20.70%)	0.636
Heartburn	55 (12.09%)	42 (9.15%)	0.149
Belching	71 (15.60%)	103 (22.44%)	0.008
Diarrhea	2 (0.44%)	3 (0.65%)	>0.999
Bitter taste	5 (1.10%)	5 (1.09%)	>0.999
Other	7 (1.54%)	8 (1.74%)	0.808
Previous eradication failures			
0	382 (83.96%)	384 (83.66%)	0.101
1	63 (13.85%)	54 (11.76%)	
≥2	10 (2.20%)	21 (4.58%)	
Family members with *H. pylori* infection			
No	54 (11.87%)	55 (11.98%)	0.799
Yes	148 (32.53%)	140 (30.50%)	
Unchecked	253 (55.60%)	264 (57.52%)	
Family history of gastric cancer	15 (3.30%)	20 (4.36%)	0.403
Antibiotic use in the past two years	269 (59.12%)	278 (60.57%)	0.656
Amoxicillin	186 (40.88%)	181 (39.43%)	0.605
Clarithromycin	24 (5.27%)	33 (7.19%)	0.231
Levofloxacin	38 (8.35%)	54 (11.76%)	0.086
Metronidazole	28 (6.15%)	35 (7.63%)	0.380
Furazolidone	4 (0.88%)	4 (0.87%)	>0.999
Tetracycline	2 (0.44%)	0 (0.00%)	0.248
Cephalosporin	134 (29.45%)	128 (27.89%)	0.601
Unknown antibiotic	42 (9.23%)	53 (11.55%)	0.251

BMI :body mass index; VA:vonoprazan -amoxicillin dual therapy.

### Eradication rates

As a result of the COVID-19 pandemic restrictions, 41 participants from the 10-day VA group and 41 participants from the 14-day VA group were no longer available for follow-up or declined to undergo ^13^C-urea breath test after receiving treatment.

The eradication rates were shown in Table 2. Multiple imputation was used to replace missing values in the ITT analysis set when certain results were not available. The eradication rate for the 10-day VA group was 88.79%, while the 14-day VA group was 92.37%. The statistical analysis showed that there was not a significant discrepancy in the eradication rates between the two groups (P = 0.064). The eradication rates of the two groups, confirmed by undergoing the ^13^C-urea breath test, were 89.14% and 92.86%. Eradication rates of 89.14% and 93.35% were observed in the per-protocol (PP) analysis.

**TABLE 2:** Difference in eradication rate between 10 d VA group and 14 d VA group.

	10d VA group	14d VA group	Different (95%CI)	P for Non-inferiority*
ITT Analysis				
Multiple imputations	88.79%	92.37%	-3.58%(-7.36%-0.20%)	0.008
Completed case	89.14%	92.86%	-3.72%(-7.65%-0.21%)	0.009
PP Analysis	89.14%	93.35%	-4.21%(-8.14%--0.27%)	0.020

*Non-inferiority margin was -10%

The non-inferiority assessment showed that the confidence intervals for the disparity in eradication rates among the groups surpassed the predetermined non-inferiority limit of −0.10 in these analysis sets. The results show that the eradication rate in the 10-day VA group was non-inferior to that of the 14-day VA group.

### Adverse events and compliance

There was no significant difference in the occurrence of adverse events between the 10-day group (20.00%, 91/455) and the 14-day group (17.86%, 82/459) (P = 0.410). The common adverse events were abdominal pain, abdominal distension, skin rash, nausea, and diarrhea (Table 3), which were mostly mild to moderate and resolved after treatment completion or withdrawal. Only one patient in the 14-day VA group was hospitalized for anti-allergic treatment due to a severe rash.

**TABLE 3:** Comparison of adverse events and compliance between 10 d VA Group and 14 d VA Group.

	10d VA group n = 455	14d VA group n = 459	P
Adverse events, n (%)			
Abdominal pain	19 (4.18)	15 (3.27)	0.468
Abdominal distension	21 (4.62)	12 (2.61)	0.105
Nausea	9 (1.98)	12 (2.61)	0.521
Diarrhea	12 (2.64)	8 (1.74)	0.355
Constipation	2 (0.44)	2 (0.44)	1.000
Acid reflux	3 (0.66)	2 (0.44)	0.992
Vomiting	4 (0.88)	6 (1.31)	0.761
Belching	1 (0.22)	4 (0.87)	0.375
Bitter taste	3 (0.66)	3 (0.65)	1.000
Headache	1 (0.22)	2 (0.44)	1.000
Skin rash	8 (1.76)	11 (2.40)	0.499
Other	7 (1.54)	8 (1.74)	0.808
Severity of adverse events, n (%)			
Mild	80 (87.91)	71 (86.59)	0.816
Moderate	11 (12.09)	10 (12.20)	
Severe	0 (0.00)	1 (1.22)	
Compliance, n (%)			
Good	396/405 (97.8)	391/406 (96.3)	0.216
Bad	9/405 (2.2)	15/406 (3.7)	

Out of the participants who underwent ^13^C-urea breath test, 9 individuals in the 10-day group and 14 individuals in the 14-day group exhibited medication adherence below 80%. Compliance did not show a statistically significant difference between the two groups (P = 0.216). The main reason for poor compliance was intolerance towards adverse events, while other reasons included busy work, patient forgetfulness, the impact of the COVID-19 epidemic, and inadequate education at enrollment.

### Risk factors affecting eradication rate

Analyze the factors influencing the eradication rate among subjects who have completed the ^13^C-urea breath test. The univariate analysis revealed that age, BMI, educational status, digestive symptoms, family history of *H. pylori* infection, and antibiotic use within the past 2 years did not significantly affect the eradication rate. Conversely, the eradication rate in the 10-day VA group could be affected by smoking and past eradication efforts. Meanwhile, in the 14-day group, variables like gender, smoking, alcohol intake, and previous eradication attempts could impact the eradication rate. Multivariate analysis showed that smoking and previous eradication failures ≥ 2 times were risk factors for VA failure (Tables 4, 5).

**TABLE 4:** Univariate and multivariate analysis of influencing factors in 10 d VA group.

		Failure	Success	OR(univariable)	OR (multivariable)
Sex	Male	22 (11.3)	173 (88.7)	—	—
	Female	22 (10.5)	188 (89.5)	1.09 (0.58-2.04, p=0.795)	0.60 (0.22-1.47, p=0.278)
Age	≤ 40	17 (9.9)	154 (90.1)	—	—
	> 40	27 (11.5)	207 (88.5)	0.85 (0.44-1.59, p=0.610)	0.78 (0.36-1.69, p=0.537)
BMI	≤ 24	30 (12.8)	204 (87.2)	—	—
	> 24	14 (8.2)	157 (91.8)	1.65 (0.86-3.30, p=0.142)	2.02 (0.97-4.42, p=0.068)
Education	High school and below	17 (11.0)	137 (89.0)	—	—
	College and above	27 (10.8)	224 (89.2)	1.03 (0.53-1.94, p=0.929)	0.92 (0.42-1.95, p=0.821)
Comorbidity	No	34 (11.6)	258 (88.4)	—	—
	Yes	10 (8.8)	103 (91.2)	1.36 (0.67-2.99, p=0.419)	1.16 (0.52-2.76, p=0.725)
Smoking	No	30 (9.1)	301 (90.9)	—	—
	Yes	14 (18.9)	60 (81.1)	0.43 (0.22-0.87, p=0.016)	0.30 (0.12-0.77, p=0.013)
Alcohol drinking	No	27 (9.6)	254 (90.4)	—	—
	Yes	17 (13.7)	107 (86.3)	0.67 (0.35-1.30, p=0.224)	0.61 (0.25-1.50, p=0.278)
Digestive symptoms	No	20 (11.0)	162 (89.0)	—	—
	Yes	24 (10.8)	199 (89.2)	1.02 (0.54-1.92, p=0.942)	1.02 (0.51-2.00, p=0.963)
Eradication history	0-1	40 (10.1)	355 (89.9)	—	—
	≥2	4 (40.0)	6 (60.0)	0.17 (0.05-0.68, p=0.008)	0.10 (0.02-0.45, p=0.002)
Family members with *H.pylori* infection	No	6 (11.8)	45 (88.2)	—	—
	Yes	14 (10.9)	114 (89.1)	1.09 (0.37-2.89, p=0.874)	1.04 (0.34-2.90, p=0.942)
	Unknown	24 (10.6)	202 (89.4)	1.12 (0.40-2.75, p=0.812)	1.10 (0.37-2.84, p=0.856)
Antibiotic use history in the past two years	No	22 (13.4)	142 (86.6)	—	—
	Yes	22 (9.1)	219 (90.9)	1.54 (0.82-2.90, p=0.176)	1.94 (0.97-3.92, p=0.061)
Adverse events	No	33 (10.1)	294 (89.9)	—	—
	Yes	11 (14.1)	67 (85.9)	0.68 (0.34-1.48, p=0.309)	0.55 (0.25-1.26, p=0.140)
Compliance	Good	43 (10.9)	353 (89.1)	—	—
	Bad	1 (11.1)	8 (88.9)	0.97(0.17-18.30,p=0.981)	1.00(0.15-19.79, p=1.000)

**TABLE 5:** Univariate and multivariate analysis of influencing factors in 14 d VA group.

		Failure	Success	OR (univariable)	OR (multivariable)
Sex	Male	19 (10.6)	161 (89.4)	—	—
	Female	10 (4.4)	216 (95.6)	2.55 (1.18-5.85, p=0.021)	1.11 (0.35-3.34, P=0.855)
Age	≤ 40	13 (7.0)	172 (93.0)	—	—
	> 40	16 (7.2)	205 (92.8)	0.97 (0.45-2.07, p=0.934)	1.25 (0.49-3.23, p=0.645)
BMI	≤ 24	17 (7.0)	227 (93.0)	—	—
	> 24	12 (7.4)	150 (92.6)	0.94 (0.44-2.06, p=0.866)	0.91 (0.39-2.19, p=0.832)
Education	High school and below	12 (7.7)	144 (92.3)	—	—
	College and above	17 (6.8)	233 (93.2)	1.14 (0.52-2.44, p=0.734)	1.36 (0.56-3.27, p=0.488)
Comorbidity	No	21 (7.2)	272 (92.8)	—	—
	Yes	8 (7.1)	105 (92.9)	1.01 (0.45-2.50, p=0.975)	1.35 (0.51-3.81, p=0.560)
Smoking	No	18 (5.2)	326 (94.8)	—	—
	Yes	11 (17.7)	51 (82.3)	0.26 (0.12-0.59, p=0.001)	0.34 (0.12-0.94, p=0.040)
Alcohol drinking	No	13 (4.5)	278 (95.5)	—	—
	Yes	16 (13.9)	99 (86.1)	0.29 (0.13-0.62, p=0.002)	0.46 (0.17-1.25, p=0.131)
Digestive symptoms	No	13 (7.7)	155 (92.3)	—	—
	Yes	16 (6.7)	222 (93.3)	1.16 (0.54-2.49, p=0.696)	0.90 (0.38-2.07, p=0.813)
Eradication history	0-1	25 (6.5)	361 (93.5)	—	—
	≥2	4 (20.0)	16 (80.0)	0.28 (0.09-1.02, p=0.031)	0.24 (0.07-0.99, p=0.033)
Family members with H.pylori infection	No	6 (11.3)	47 (88.7)	—	—
	Yes	10 (8.1)	114 (91.9)	1.46 (0.47-4.15, p=0.491)	1.21 (0.36-3.81, p=0.745)
	Unknown	13 (5.7)	216 (94.3)	2.12 (0.71-5.67, p=0.147)	2.13 (0.65-6.49, p=0.190)
Antibiotic use history in the past two years	No	9 (5.8)	147 (94.2)	—	—
	Yes	20 (8.0)	230 (92.0)	0.70 (0.30-1.55, p=0.398)	0.86 (0.34-2.06, p=0.736)
Adverse events	No	22 (6.4)	321 (93.6)	—	—
	Yes	7 (11.1)	56 (88.9)	0.55 (0.23-1.44, p=0.189)	0.44 (0.17-1.22, p=0.096)
Compliance	Good	26 (6.6)	365 (93.4)	—	—
	Bad	3 (20.0)	12 (80.0)	0.28 (0.08-1.31, p=0.064)	0.38 (0.10-1.95, p=0.198)

### Cost-effectiveness analysis results

#### Fundamental analysis

Cost-effectiveness was used for economic evaluation. The eradication rates of the 10-day group and the 14-day group were 89.14% and 93.35%, respectively, and the difference in eradication rates was statistically significant (P = 0.037). The cost of medication for the 10-day group was 69.3 × 3 + 1.90 × 5 = 217.4 yuan, and the cost of medication for the 14-day group was 69.3 × 4 + 1.90 × 3 = 290.5 yuan; Diagnosis and treatment cost = registration fee+breath test C13 = 12 + 150 = 162 yuan; The specific cost information was shown in Table 6. The cost-effectiveness ratio of the 10-day group was 426.30 yuan, the cost-effectiveness ratio of the 14-day group was 485.27 yuan, and the incremental cost-effectiveness ICER was 1680.23 yuan, as shown in Table 7.

**TABLE 6:** Breakdown and total costs for the 10-day and 14-day groups.

	10d VA group	14d VA group
Drug cost (yuan)	217.4	290.5
Examination cost (yuan)	162	162
ADR cost (yuan)	0.63	0.59
Total cost (yuan)	380 ± 4.74	453 ± 6.09

ADR: adverse events.

**TABLE 7:** Cost-effectiveness between the 10-day group and the 14-day group.

	10d VA group	14d VA group
Eradication rate	89.14%	93.35%
Total cost (yuan)	380	453
CER (yuan)	426.30	485.27
ICER (yuan)	1680.23	

CER: cost-effectiveness ratio; ICER: incremental cost-effectiveness ratio.

#### Sensitivity analysis

**FIGURE 2:**
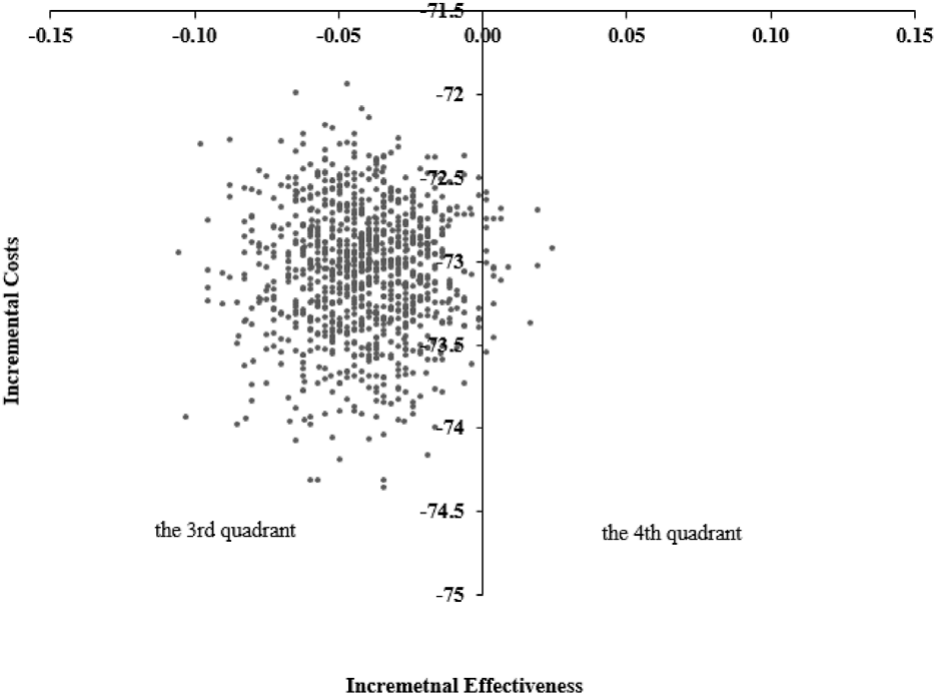
Scatter plot of incremental cost-effectiveness probability sensitivity analysis between 10-day and 14-day groups of *Helicobacter* pylori-infected patients (n = 1000) (The horizontal axis represented the incremental effect compared to the 14-day group (eradication rate; 10-day group effect −14-day group effect), and the vertical axis represents the incremental cost compared to the 14-day group (10-day group cost −14-day group cost). The third quadrant indicated that the 10-day group has poor performance and low cost, while the fourth quadrant indicated that the 10-day group was an absolutely advantageous solution (with good performance and low cost)).

**FIGURE 3:**
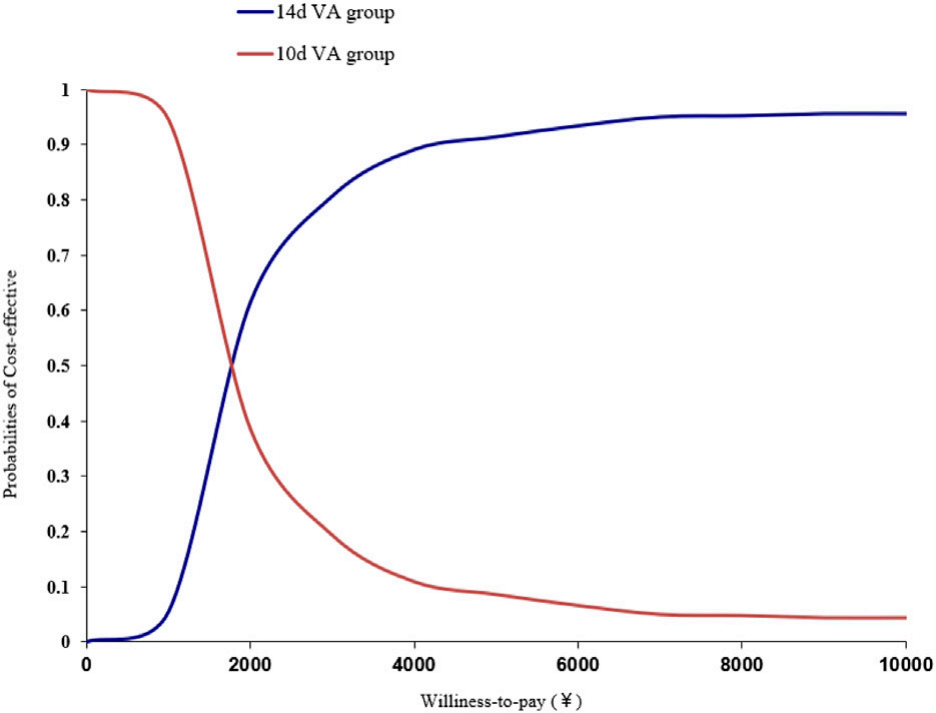
Cost-effectiveness acceptable curve (CEAC) analysis of probability sensitivity analysis of *Helicobacter pylori* patients at 10 Days compared to 14 Days.

The scatter plot of incremental cost-effectiveness was shown in Figure 2. As shown in Figure 2, most of the points were distributed in the third quadrant, indicating that the 10-day group has lower effectiveness and cost; A few points were distributed in the fourth quadrant, indicating that the 10-day group had better results and lower costs. When the cost-effectiveness analysis results were in the third quadrant, threshold assisted decision-making could be used. Draw an acceptable cost-effectiveness curve based on the percentage of estimated points below the willingness-to-pay threshold (Figure 3). As shown in Figure 3, as the willingness-to-pay threshold increases, the economic probabilities of the 10-day and 14-day groups change. The intersection point in the figure is (1742, 50%), which indicated that when the willingness-to-pay threshold was 1742 yuan, the probability of the two schemes having economic viability was the same. When the decision maker’s willingness-to-pay threshold was below 1742 yuan (on the left side of the intersection), the probability of the 10-day group being more cost-effective remains above 50%. When the decision maker’s willingness-to-pay threshold exceeds 1742 yuan (on the right side of the intersection), the 14-day group had a higher probability of being more cost-effective than the 10-day group. The specific results were shown in Figure 3.

## Discussion

The latest treatment guidelines for *H. pylori* in China were released in 2022. The guideline recommended the classic bismuth quadruple therapy(BQT) as the mainstream first-line treatment for *H. pylori* in China and also proposed the emerging high doses of amoxicillin (HDDT) as a first-line eradication solution for *H. pylori* (Zhou et al., 2022). The previous dual regimen of PPI combined with amoxicillin had achieved good results in China. Currently, the recommended dosage for this regimen is high-dose amoxicillin (≥3 g/d) and double-dose PPI for 14 days.

The new acid suppressant, vonoprazan, reversibly inhibits gastric H+, K+-ATPase, exhibiting a rapid onset of action, ensuring both efficacy and durability and operating independently of acid activation. It maintains stability under acidic conditions and remains unaffected by CYP2C19 gene polymorphism (Miftahussurur et al., 2020). After being taken orally, it can achieve peak plasma levels within 1.5–2.0 h and effectively inhibit acid production for a duration of 24 h (Echizen, 2016). The vonoprazan-amoxicillin(VA) dual therapy was first used in *H. pylori* eradication studies in Japan and achieved a good eradication rate (Murakami et al., 2016). In Japan, a low-dose 7-day VA treatment (amoxicillin 750 mg bid) has shown a similar effect to the 7-day vonoprazan, amoxicillin, clarithromycin triple therapy, with an ITT analysis eradication rate ranging from 85% to 93% (Suzuki et al., 2020; Furuta et al., 2020). However, research in Europe and the US revealed that the success rate of the 14-day high-dose VA treatment was only 78.5%, falling short of expectations (Chey et al., 2022). These variations could be attributed to disparities in ethnicity, physique, various *H. pylori* types, and the way CYP3A4 metabolizes substances. Therefore, there was an urgent need to study and determine the treatment course and drug dosage of VA regimen in China. Han et al. research indicated that the success rate of the 10-day VA treatment was 91.4%, proving its effectiveness compared to the 14-day rabeprazole and high-dose amoxicillin treatment (Han et al., 2023). Yan et al. demonstrated that the success rate of eliminating the infection with the 10-day VA was 90.8%, proving to be just as effective as the 14-day BQT (Yan et al., 2023). Therefore, The 10-day VA treatment may have a satisfactory eradication effect. However, these researches lacked a comparison between the treatment courses of the VA regimen. Therefore, we designed this randomized controlled multicenter, large-sample clinical study to compare the effectiveness, safety, and economy of the 10-day and the 14-day VA regimen.

Our study revealed that the eradication rate in the 10-day VA group was non-inferior to that of the 14-day VA group, which may be due to the synergistic effect of amoxicillin in the strong low-acid environment composed of vonoprazan, which fully exerts its bactericidal effect. Nevertheless, the eradication rate of the 10-day VA in our study dropped below the target of 90%, failing to reach the optimal eradication rate for the first-line treatmen (Federico et al., 2014). Consequently, in clinical settings where initial treatment is crucial, the 14-day VA regimen may offer advantages (Zhong et al., 2022). In terms of economic evaluation, the incremental cost-effectiveness ratio (ICER) of the 10-day and 14-day groups was 1680.23. Probability sensitivity analysis showed that the 10-day group was more cost-effective, but as the willingness-to-pay threshold increased to 1742 yuan, the probability of the 14-day group being more cost-effective was greater than that of the 10-day group. In addition, analysis revealed that smoking was a factor that impacted the success rate of the VA treatment regimen. The discovery was linked to the amount of smoking and the act of smoking while undergoing treatment. Smoking can reduce blood flow and mucus production in the stomach, hindering the antibiotics' ability to reach the stomach lining effectively. Additionally, it can stimulate gastric acid secretion and alter the activity of CYP450 isoenzymes (Suzuki et al., 2006). Yu et al.'s meta-analysis also indicated that smoking reduces the eradication rate of *H. pylori* (Yu et al., 2022). The result of our study was consistent with the conclusions of the meta-analysis mentioned above.

Furthermore, this study included patients with previous eradication failure to assess the effectiveness of the VA therapy in rescue therapy. The subgroup analysis showed that the eradication rate for participants with at least two prior eradication failures was below 90%, a lower rate than the study by Gao et al. (Gao et al., 2022). This difference may be due to varying numbers of previous eradication failures and individual variations in *H. pylori* resistance. And due to the small number of subjects who failed eradication more than twice in the two groups, no statistical difference comparison was conducted. Further research is needed to expand the sample size. Therefore, further studies are needed to confirm the effectiveness of VA therapy in rescue treatment, especially in patients who have failed multiple eradication attempts.

We must acknowledge some limitations of the present study. Initially, not every subject received ^13^C-urea breath test following eradication. Despite utilizing the multiple imputation technique to estimate the eradication rate of the ITT analysis group, discrepancies persisted compared to the actual data. Second, no antibiotic sensitivity test or genotype resistance test was conducted in this study, and the impact of amoxicillin resistance and host CYP3A4 genotype metabolism on the eradication effect could not be further explored. Finally, this study only utilized a high dose of amoxicillin, further research on a lower dosage of amoxicillin is required.

In conclusion, the 10-day VA treatment was no-inferior to the 14-day in *H. pylori* eradication and the 10-day VA group was more cost-effective. However, due to the eradication rate not reaching 90% with the 10-day VA treatment, the 14-day VA treatment was still recommended as the first-line treatment in China. For patients who cannot tolerate a 14-day course of treatment, it is recommended to take it orally for at least 10 days to ensure an eradication rate of around 89%. In addition, a history of smoking or previous eradication failure is associated with the failure of VA therapy, and these patients should carefully consider the specific circumstances when choosing VA therapy.

## Data Availability

The original contributions presented in the study are included in the article/supplementary material, further inquiries can be directed to the corresponding author.
