# Supplementary figures and images for: Efficacy and cost-effectiveness analysis of 10-day versus 14-day eradication of Helicobacter pylori infection with vonoprazan amoxicillin: a prospective, multicenter, randomized controlled trial
Source: Front Pharmacol. 2025 Mar 24;16:1543352. doi: 10.3389/fphar.2025.1543352 (PMC11973372; doi:10.3389/fphar.2025.1543352)

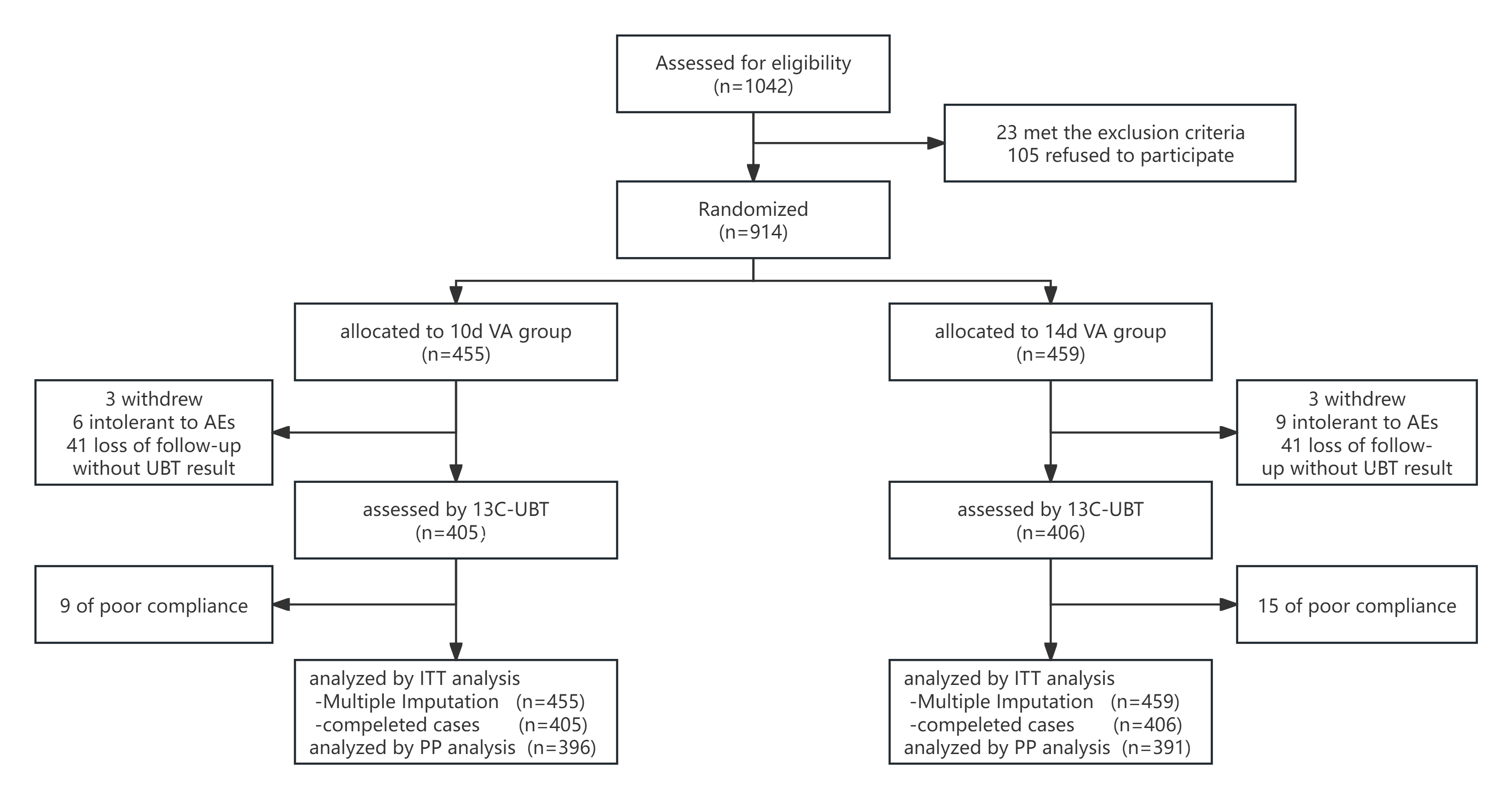

Supplement: Supplementary file 1 [file Image1.png]
